# A39 DEVELOPMENTALLY REGULATED CECAL CONTENT MICRO-RNAS CORRELATE WITH MATURING MICROBIOTA GENES AND FUNCTIONS IN JUVENILE MICE

**DOI:** 10.1093/jcag/gwad061.039

**Published:** 2024-02-14

**Authors:** C Cuinat, A Taibi, J Tremblay, G Gargari, S Guglielmetti, T Tompkins, E Comelli

**Affiliations:** University of Toronto Department of Nutritional Sciences, Toronto, ON, Canada; University of Toronto Department of Nutritional Sciences, Toronto, ON, Canada; Energy, Mining and Environment, National Research Council Canada, Montréal, QC, Canada; Department of Food, Environmental and Nutritional Sciences, University of Milan, Milan, Italy; Department of Food, Environmental and Nutritional Sciences, University of Milan, Milan, Italy; . Rosell® Institute for Microbiome and Probiotics, Montréal, QC, Canada; University of Toronto Department of Nutritional Sciences, Toronto, ON, Canada

## Abstract

**Background:**

The gut microbiome establishment in early life is critical to life-long health and influenced by the maternal gut ecosystem. MicroRNAs (miRNAs) have emerged as a new player in host-microbiota interaction due to their regulatory effect on bacterial genes. We previously found that maternal supplementation with a probiotic mix of *Lacticaseibacillus rhamnosus* R0011 and *Lactobacillus helveticus* R0052 supported the maturation of microbial metabolic activity and altered the cecal content miRNA profile in juvenile mice.

**Aims:**

To investigate the relationship between cecal content miRNAs and inferred microbiota genes and functions in early life and identify potential miRNA-bacterial gene targets.

**Methods:**

We generated 16S rRNA gene sequencing and NanoString nCounter® data from the cecal content of 14, 21, and 36-days-old C57BL/6 mice born to dams receiving or not probiotic-supplemented water since preconception. Taxa contributions to bacterial enzyme-encoding genes and pathways were inferred with PICRUSt2. Time or group-altered genes, pathways, and miRNAs were identified with DESeq2. Spearman correlations were performed between miRNAs and bacterial genes or pathways (n= 3-5 male offspring/PND/group) and significant correlations (q ampersand:003C 0.05) were visualized with NAViGaTOR. Potential miRNA binding sites on bacterial genes were investigated using the ViennaRNA Package.

**Results:**

Time-associated miR-433 positively correlated with two time-altered genes involved in the TCA and glyoxylate cycles. Group-associated miR-691 was positively correlated with genes related to tRNA charging pathway, pyruvate fermentation to acetate and lactate, and amino acids biosynthesis. Additionally, miR-691 was negatively correlated with six group-altered genes involved in myo-inositol degradation. We identified potential miRNA binding sites (total free energy of binding ampersand:003C -10 kcal/mol) for miR-691 with genes involved in pyruvate fermentation, lysine biosynthesis, and myo-inositol degradation, and for miR-433 with genes annotated to the TCA and glyoxylate cycles.

**Conclusions:**

Host miRNAs correlate with bacterial pathways and genes maturing during the weaning transition, highlighting their potential to regulate microbial metabolic activity. Maternal probiotics supplementation may accelerate the development of microbial metabolic pathways through epigenetic mechanisms, independently of weaning dietary shift. Correlations identified include energy production pathways and may become clinical targets in infants with delayed maturation of the intestinal ecosystem.

**Funding Agencies:**

NSERC

